# Presynaptic dopaminergic imaging as a neurodegeneration staging biomarker in the alpha-synucleinopathy continuum

**DOI:** 10.1007/s00259-026-07834-1

**Published:** 2026-04-01

**Authors:** Dario Arnaldi, Pietro Mattioli, Beatrice Orso, Stefano Raffa, Francesco Lanfranchi, Federico Massa, Alex Iranzo, Andres Perissinotti, Aida Niñerola-Baizán, Carles Gaig, Monica Serradell, Angelica Montini, Gerard Mayà, Amaia Muñoz-Lopetegi, Claudio Liguori, Mariana Fernandes, Fabio Placidi, Agostino Chiaravalloti, Karel Šonka, Petr Dušek, David Zogala, Jiri Trnka, Bradley F. Boeve, Toji Miyagawa, Val J. Lowe, Tomoyuki Miyamoto, Masayuki Miyamoto, Monica Puligheddu, Michela Figorilli, Alessandra Serra, Michele T. Hu, Johannes C. Klein, Frederik Bes, Dieter Kunz, Valérie Cochen De Cock, Delphine De Verbizier, Giuseppe Plazzi, Elena Antelmi, Fabio Pizza, Michele Terzaghi, Irene Bossert, Kristína Kulcsárová, Valentina Garibotto, Nicolas Nicastro, Aurelien Lathuilière, Laura Bonanni, Silvia Morbelli, Monica Roascio, Gabriele Arnulfo, Matteo Pardini

**Affiliations:** 1 Department of Neuroscience (DINOGMI), University of Genoa, Genoa, Italy; 2 IRCCS Ospedale Policlinico San Martino, Genoa, Italy; 3 Neurology Service, Sleep Disorder Centre, Hospital Clínic Barcelona, Universitat de Barcelona, IDIBAPS, CIBERNED: CB06/05/0018-ISCIII, Barcelona, Spain; 4 Nuclear Medicine Service, Hospital Clínic Barcelona - IDIBAPS, CIBER-BBN, ISCIII, Barcelona, Spain; 5 Department of Systems Medicine, University of Rome Tor Vergata, Rome, Italy; 6 Sleep Medicine Center, Neurology Unit, University Hospital of Rome Tor Vergata, Rome, Italy; 7 Department of Biomedicine and Prevention, University of Rome Tor Vergata, Rome, Italy; 8 IRCCS Neuromed, Pozzilli, Italy; 9 Department of Neurology, Center of Clinical Neuroscience, First Faculty of Medicine, Charles University and General University Hospital, Prague, Czech Republic; 10 Institute of Nuclear Medicine, First Faculty of Medicine, Charles University and General University Hospital, Prague, Czech Republic; 11 Department of Neurology, Mayo Clinic, Rochester, MN USA; 12 Department of Radiology, Mayo Clinic, Rochester, MN USA; 13 Department of Neurology, Dokkyo Medical University Saitama Medical Center, Saitama, Japan; 14 Center of Sleep Medicine, Dokkyo Medical University Hospital, Tochigi, Japan; 15 Sleep Disorder Center, Department of Public Health and Clinical and Molecular Medicine, University of Cagliari, Cagliari, Italy; 16 Nuclear Medicine Unit, Department of Medical Science and Public Health, University of Cagliari, Cagliari, Italy; 17 Division of Neurology, Nuffield Department of Clinical Neurosciences, Oxford University, Oxford, UK; 18 Clinic of Sleep & Chronomedicine, St. Hedwig-Hospital, Berlin, Germany; 19 Institute of Physiology, Sleep Research & Clinical Chronobiology, Charité– Universitätsmedizin Berlin, Berlin, Germany; 20 Sleep and neurology department, Beau Soleil Clinic, Montpellier, France; 21 EuroMov Digital Health in Motion, Univ Montpellier, IMT Mines Ales, Montpellier, France; 22 Nuclear Medicine Unit, University hospital of Montpellier, Montpellier, France; 23 IRCCS Istituto delle Scienze Neurologiche di Bologna, Bologna, Italy; 24 Department of Biomedical, Metabolic and Neural Sciences, University of Modena and Reggio-Emilia, Modena, Italy; 25 DIMI Department of Engineering and Medicine of Innovation, University of Verona, Verona, Italy; 26 Dipartimento di Scienze Biomediche e Neuromotorie (DIBINEM), Università di Bologna, Bologna, Italy; 27 Sleep Medicine and Epilepsy Unit, IRCCS Mondino Foundation, Pavia, Italy; 28 Department of Brain and Behavioral Sciences, University of Pavia, Pavia, Italy; 29 Nuclear Medicine Unit, ICS Maugeri SpA SB IRCCS, Pavia, Italy; 30 Department of Neurology, P. J. Safarik University, Kosice, Slovak Republic; 31 Department of Neurology, University Hospital of L. Pasteur, Kosice, Slovak Republic; 32 Department of Clinical Neurosciences, P. J. Safarik University, Kosice, Slovak Republic; 33 Geneva University Hospitals and Geneva University, Geneva, Switzerland; 34 Department of Medicine and Aging Sciences, University G. d’Annunzio of Chieti- Pescara, Chieti, Italy; 35 Nuclear Medicine Unit, AOU Citta’ della Salute e della Scienza di Torino, Turin, Italy; 36 Department of Medical Sciences, University of Turin, Turin, Italy; 37 Department of Informatics, Bioengineering, Robotics and System Engineering (DIBRIS), University of Genoa, Genoa, Italy; 38 Child Neuropsychiatry Unit, IRCCS Istituto Giannina Gaslini Full Member of the ERN EpiCARE, Genoa, Italy

**Keywords:** SPECT, PD, DLB, RBD, Neurodegeneration, Staging

## Abstract

**Purpose:**

To define how dopamine transporter (DaT) SPECT can be used to stage neurodegeneration in neuronal alpha-synucleinopathy patients at the individual level.

**Methods:**

This is an international multicenter study involving 1067 subjects (mean age 69.8 ± 8.7 years; 63.2% males) who underwent DaT-SPECT as a neurodegeneration biomarker. We enrolled 277 controls, 400 patients with idiopathic/isolated REM sleep behavior disorder (iRBD), representing the prodromal alpha-synucleinopathy stage, and 390 patients with an overt stage, including 175 with overt Parkinson’s disease (oPD) and 215 with overt dementia with Lewy bodies (oDLB). iRBD patients were followed over time and were stratified as non-converters (ncRBD, *n* = 232) or converters (cRBD, *n* = 168). The ability of DaT-SPECT to stage the neuronal alpha-synucleinopathy continuum was evaluated using forward stepwise logistic regression models, assuming that this stratification reflects progressive neurodegeneration stages (controls, ncRBD, cRBD, and overt PD/DLB).

**Results:**

The combination of the most affected putamen and the least affected caudate best staged patients across the continuum (*p* < 0.001). Our data suggest that the most affected putamen z-scores can be used to define three levels of neurodegeneration, such as undetected (above − 1), moderate (between − 1 and − 2), and severe (below − 2). Cox regression analysis in iRBD patients showed that these cutoffs predicted phenoconversion (hazard ratios 3.10–5.03), outperforming clinical risk metrics (MDS-UPDRS-III, MMSE, and hyposmia) for overall and motor-predominant phenoconversion.

**Conclusion:**

DaT-SPECT z-score thresholds provide a ready-to-use three-tier staging system for alpha-synucleinopathies, enabling objective assessment of neurodegeneration severity and phenoconversion risk at the individual level.

**Supplementary Information:**

The online version contains supplementary material available at 10.1007/s00259-026-07834-1.

## Introduction

Parkinson disease (PD) and dementia with Lewy bodies (DLB) are included under the umbrella terms neuronal alpha-synucleinopathies or Lewy body disorders and are characterized by a long prodromal period before the emergence of overt clinical syndromes. New frameworks have been proposed, providing both a biological definition and a staging system for the prodromal to overt neuronal alpha-synucleinopathy continuum [1, 2]. The current proposals endorse the use of presynaptic dopaminergic imaging as the most ready neurodegeneration biomarker [1, 2]. However, clear operational instructions regarding how presynaptic dopaminergic imaging should be employed have not yet been provided, nor clear cut-off values that can be used at the individual level. This research and clinical gap jeopardizes the applicability of any definition and staging system in clinical practice.

The most commonly used presynaptic dopaminergic imaging technique is dopamine transporter single-photon emission computed tomography with [^123^I]FP-CIT (DaT-SPECT). DaT-SPECT is a well-established and methodologically robust nigro-striatal dopaminergic neurodegeneration biomarker, and it is approved for clinical practice in most high-income countries. Acquisition and reconstruction protocols are standardized and described in international guidelines [3]. Literature data suggest that DaT-SPECT imaging, if properly semi-quantified, can be used at the individual level in both prodromal [4, 5] and overt [6–8] alpha-synucleinopathy stages. However, clear cut-off values to be used for staging patients across the prodromal to overt neuronal alpha-synucleinopathy continuum are still missing, as well as robust data on which basal ganglia regions are most suitable for assessing neurodegeneration. For example, while the most affected putamen has been suggested as the best region in the prodromal stage [4], the least affected putamen showed more robust results in monitoring disease progression in the overt stage [9]. Thus, a combination of DaT-SPECT features may be needed to assess patients across the whole prodromal-to-overt continuum.

Finally, although current proposals for a biological definition of neuronal alpha-synucleinopathies suggest that PD and DLB should be included in the same disease spectrum, nigro-striatal dopaminergic dysfunction may have disease-specific features. PD patients usually show more severe alterations, while DLB patients may have more heterogeneous DaT-SPECT findings, often described as a balanced loss (or “weak comma”) due to diffuse reduction in uptake [10], but also normal scans [11], especially in DLB patients without or with mild parkinsonism [12]. Therefore, it is crucial to investigate the specific characteristics of DaT-SPECT abnormalities in both motor-predominant (parkinsonism-first) and cognitive-predominant (dementia-first) pathways.

This is a large, multicentric, longitudinal study aimed at defining how DaT-SPECT should be used as a neurodegeneration staging biomarker at the individual level across the prodromal-to-overt neuronal alpha-synucleinopathy continuum.

## Materials and methods

### Subjects

This is a retrospective, longitudinal, international multicentre study including 15 centres worldwide (Barcelona, Berlin, Bologna, Cagliari, Chieti, Dokkyo, Geneva, Genoa, Kosice, Montpellier, Oxford, Pavia, Prague, Rochester and Rome Tor Vergata, Table S1), involving 1067 subjects (69.8 ± 8.7 years at DaT-SPECT, 63.2% males). In detail, we enrolled 277 controls without definite neurological or psychiatric diseases (68.3 ± 9.0 years, 50.2% males), who underwent DaT-SPECT for excluding PD (including but not limited to essential tremor), 400 patients with idiopathic/isolated REM sleep behaviour disorder (iRBD, 68.9 ± 6.9 years, 75.2% males), representing the prodromal alpha-synucleinopathy stage, and 390 patients with an overt stage (71.7 ± 9.9 years, 60.0% males), including 175 drug naïve, overt PD (oPD, 68.7 ± 8.6 years, 60.0% males) and 215 drug naïve, overt DLB (oDLB, 74.2 ± 10.l2 years, 60.0% males). All clinical diagnoses followed current international criteria [13–15], including overnight polysomnography in all iRBD patients. The iRBD group was used in a previous multicentric study [4].

All iRBD patients were followed over time (40.9 ± 30.4 months from DaT-SPECT) to investigate phenoconversion, that is, the emergence of overt PD [15] or DLB [14]. The iRBD group was retrospectively stratified, according to the follow-up outcome, as non-converters (ncRBD, *n* = 232, 67.6 ± 7.1 years, 78.4% males) if they were still free from overt parkinsonism or dementia at last follow-up, or as converters if parkinsonism or dementia were documented up to the last follow up (cRBD, *n* = 168, 70.8 ± 6.1 years, 70.8% males). Moreover, the cRBD group was further stratified as prodromal PD (pPD, *n* = 94, 69.7 ± 5.8 years, 61.7% males) or prodromal DLB (pDLB, *n* = 74, 72.1 ± 6.1 years, 82.4% males), according to the final diagnosis.

We assumed that this stratification reflects patients’ neurodegeneration stages along the alpha-synucleinopathy continuum, in other words, that prodromal patients are likely to be at an earlier neurodegeneration stage than overt patients. Moreover, non-converters RBD (i.e., those who did not develop parkinsonism or dementia in the short-term) likely have a milder degree of neurodegeneration as compared with converters RBD (i.e., those who phenoconverted on a short-term). Notably, the follow-up time of the ncRBD group (40.4±30.6 months) is comparable to the phenoconversion time of the cRBD (41.5±30.3 months), suggesting that these two groups are in different neurodegeneration stages, not strictly related to the time of diagnosis or follow-up. We acknowledge that this approach may not be the best clinical staging system, but it follows the most used and widely accepted categorization of alpha-synucleinopathy patients that identifies patients in a prodromal stage when overt parkinsonism or dementia is absent, and patients phenoconverting to the overt stage when clinical criteria for PD and DLB are fulfilled.

**Fig. 1:**
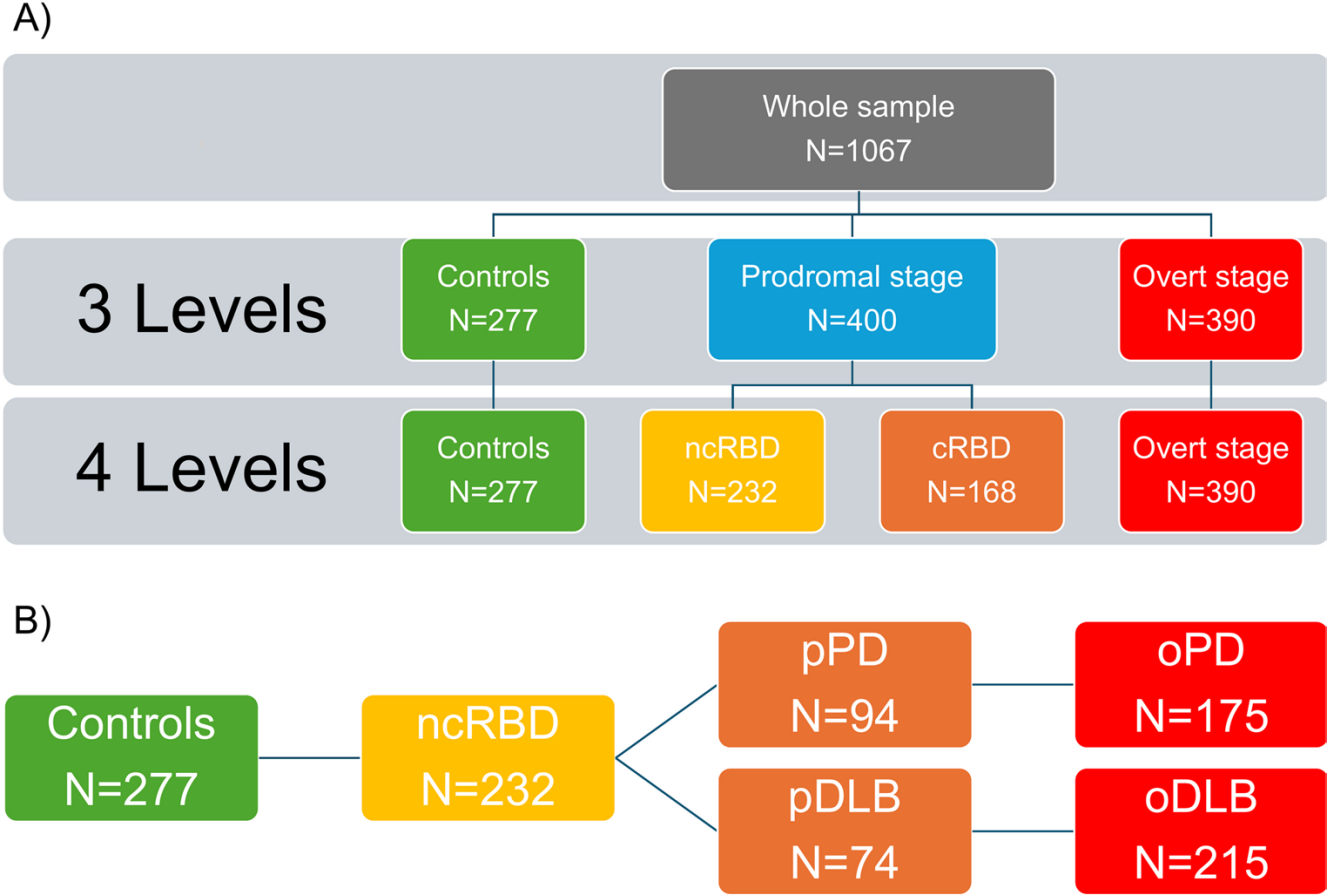
Study sample clinical diagnosis and stratifications. (**A**) Whole sample stratification, with increasing degree of granularity, using 3-staging levels (controls, prodromal and overt stages) and 4-staging levels (controls, non-converters RBD [ncRBD], converters RBD [cRBD], and overt stage). (**B**) Prodromal to overt neuronal alpha-synucleinopathy continuum, divided in a motor-predominant pathway (control, ncRBD, prodromal PD [pPD], and overt PD [oPD]) and a cognitive-predominant pathway (control, ncRBD, prodromal DLB [pDLB], and overt DLB [oDLB])

Following this assumption, we identified three (controls, prodromal, and overt stages) and four (controls, ncRBD, cRBD, and overt stage) levels of granularity, assuming that those levels should be representative of the progressive neurodegeneration stages (Fig. 1A). Moreover, we also split the whole cohort into a motor-predominant pathway (Fig. 1B, controls, ncRBD, pPD, and oPD) and a cognitive-predominant pathway (Fig. 1B, controls, ncRBD, pDLB, and oDLB) to investigate the different clinical trajectories.

For this study, we did not enroll patients with multiple system atrophy (MSA) because of the different physiopathological underlying mechanisms, even though we cannot exclude that some of the ncRBD patients may develop MSA in the future.

Motor function was assessed with the Movement Disorder Society Unified PD Rating Scale, motor section (MDS-UPDRS-III) [16]; scores from the 1987 version of the UPDRS-III were converted into MDS-UPDRS-III [17] scores, which were used for statistical analysis. Global cognition was assessed using the Mini-Mental State Examination (MMSE) test [18]. Montreal Cognitive Assessment (MoCA) [19] scores were converted into MMSE scores [20], and only the MMSE scores were used for statistical analysis. Olfaction was assessed using the 40-item University of Pennsylvania Smell Identification Test [21], Sniffin’ Sticks 16-item odor identification test [22], or Odor Stick Identification Test for Japanese [23].

### Ethical approval

All participants (or their proxies, as appropriate) signed an informed consent form in compliance with the Declaration of Helsinki of 1975. Ethics approval was obtained from the local institutional review boards in all participating centers, and the coordinating center’s institutional board also approved the study (184REG2017).

## [^123^I]-Ioflupane SPECT ([^123^I]FP-CIT-SPECT)

All subjects underwent DaT-SPECT as a marker of nigrostriatal dopaminergic functioning. Images were acquired after i.v. administration of 156.7 ± 26.2 MBq of [^123^I]FP-CIT (DaTSCAN, GE Healthcare, Little Chalfont, Buckinghamshire, UK) according to international guidelines [3, 24] DaT-SPECT was semi-quantified using DaTQUANT™ V2 software (GE Healthcare), as detailed elsewhere [4, 25] To compute the age and sex adjusted z-scores for all basal ganglia features in all subjects, we used a dataset based on 118 healthy volunteers (no first-degree blood relatives affected by PD; 73 men and 45 women, aged 31 to 84 years) belonging to the PPMI database (more details can be found at https://www.ppmi-info.org), already included in DaTQUANT™. We previously demonstrated that, by using this approach, the center effect is negligible [4]; thus, we did not apply a center effect correction in the present study.

DaT-SPECT data were flipped to have the most affected hemisphere (MAH) and the least affected hemisphere (LAH) (i.e., the hemisphere with the highest/lowest value between left and right, respectively) on the same side for all patients for statistical analysis. In 51.7% of the subjects, the MAH was on the left.

### Statistical analysis

A first descriptive analysis was performed by comparing the study groups across the 3-staging levels (controls, prodromal, and overt stage) and the 4-staging levels (controls, ncRBD, cRBD, and overt stage). Between-group differences were assessed using the univariate analysis of variance (ANOVA) for normally distributed continuous variables, the Kruskal-Wallis test for non-normally distributed continuous variables, and the chi-square test for categorical variables.

To investigate the association between DaT-SPECT features (MAH/LAH putamen, caudate, and putamen/caudate ratios) and the neurodegeneration stages along the alpha-synucleinopathy continuum, we applied two forward stepwise logistic regression models, using both the 3-staging levels and the 4-staging levels stratification as the dependent variable, respectively, and the DaT-SPECT features as the independent variables. Subsequently, two additional forward stepwise logistic regression models were applied to investigate the association between DaT-SPECT features and both the motor-predominant and the cognitive-predominant pathways.

## Results

### Descriptive analysis

Tables S2, S3, S4, and S5 summarize the main clinical and demographic data of the whole cohort, stratified according to 3-staging levels (controls, prodromal, and overt stages) and 4-staging levels (controls, ncRBD, cRBD, and overt stage), as well as the motor-predominant and the cognitive-predominant pathways.

### DaT-SPECT features across the prodromal to overt neuronal alpha-synucleinopathy continuum

**Fig. 2:**
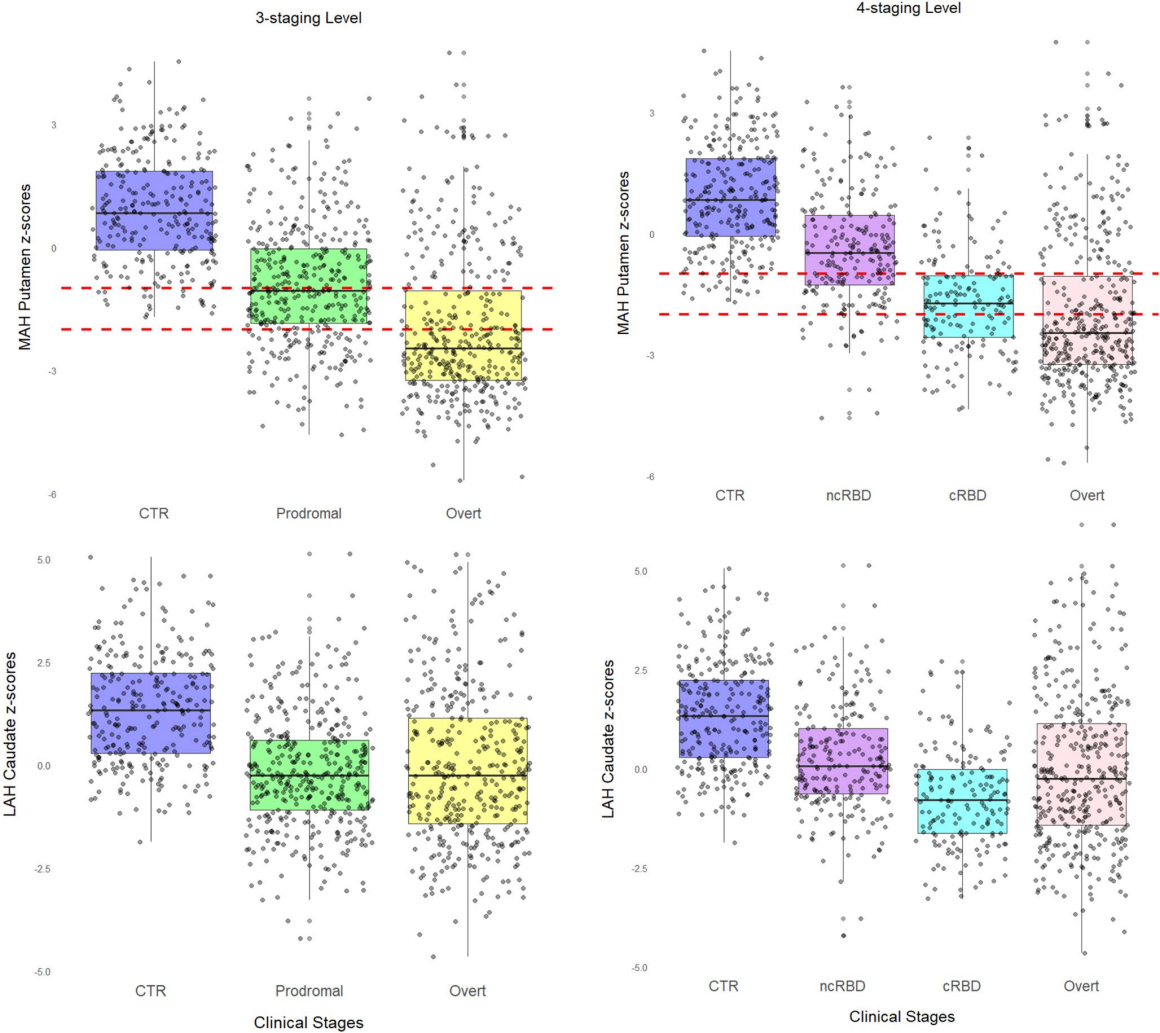
Presynaptic dopaminergic imaging features across the prodromal to overt neuronal alpha-synucleinopathy continuum. Box plot of the output of the two built models, representing the results for the 3-staging levels (left pane columns: controls, prodromal and overt stages) and the 4-staging levels (right pane columns: controls, non-converters RBD [ncRBD], converters RBD [cRBD], and overt stage) models. The upper row shows the most affected putamen z-scores, and the dashed red lines represent the − 1 and − 2 z-scores cut-offs, respectively. The lower row shows the least affected caudate z-scores in both models

Both the 3-staging and the 4-staging levels models showed a significant association between DaT-SPECT features and the a priori defined clinical stages (*p* < 0.001). Both models demonstrated that the combination of MAH putamen (coefficient 1.52, standard error, SE 0.10; odds ratio, OR 4.57, 95% confidence interval, CI 3.80–5.55) and LAH caudate (coefficient − 1.17, SE 0.10; OR 0.31, CI 0.26–0.37) was the most effective in predicting clinical stages (Fig. 2, Tables S6 and S7).

The MAH putamen z-score distributions showed progressive decline across stages. Among subjects with z-scores below − 1, 75.6% of cRBD (high short-term phenoconversion risk) fell into this category compared to only 35.0% of ncRBD (lower risk), suggesting this threshold effectively stratifies prodromal patients by phenoconversion risk. The more stringent z-score below − 2 threshold captured 61.8% of overt patients, indicating more advanced neurodegeneration, though 38.2% of overt patients remained above this threshold, with greater heterogeneity observed in the cognitive-predominant pathway (see below).

**Fig. 3:**
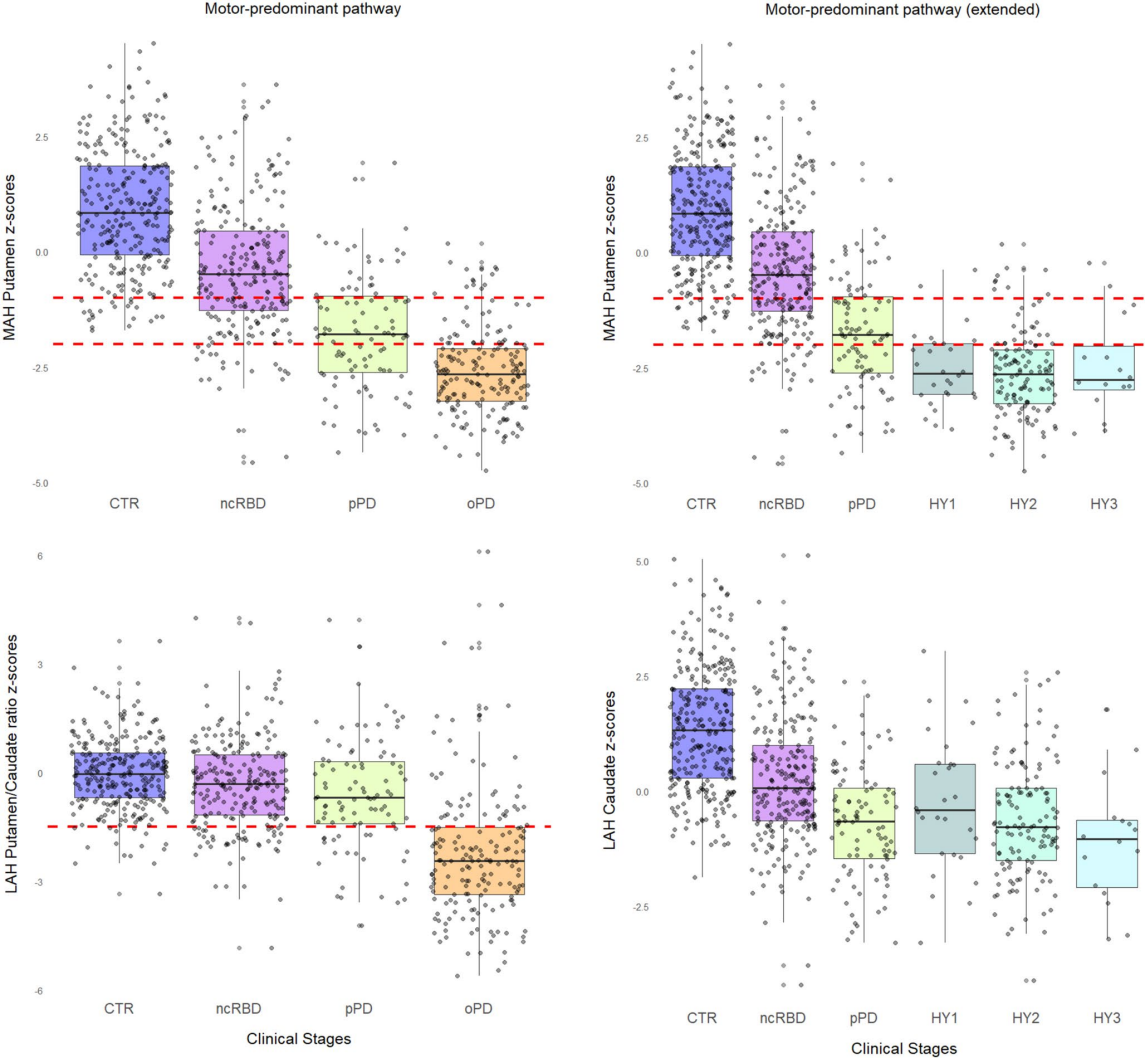
Presynaptic dopaminergic imaging features across the motor-predominant pathway. Box plot of the output of the two built models, representing the results for the motor-predominant pathway (left pane columns: controls [CTR], non-converters RBD [ncRBD], prodromal PD [pPD], and overt PD [oPD]) and the motor-predominant extended pathway (right pane columns: controls, non-converters RBD, prodromal PD, and overt PD with Hoehn and Yahr scale score of 1, 2 and 3, respectively). The upper row shows the most affected putamen z-scores for both models, and the dashed red lines represent the − 1 and − 2 z-scores cut-offs, respectively. The lower left graph shows the least affected putamen/caudate z-scores for the motor-predominant pathway, and the dashed red line represents the − 1.5 z-score cut-off. The lower right graph shows the least affected caudate z-scores for the motor-predominant extended pathway

The model assessing the association between DaT-SPECT features and the motor-predominant pathway (Fig. 3, Table S8) revealed that the best combination for predicting clinical stages (*p* < 0.001) includes the MAH putamen (coefficient 1.12, SE 0.11; OR 3.08, CI 2.50–3.88) and the LAH putamen/caudate ratio (coefficient 0.61, SE 0.09; OR 1.84, CI 1.55–2.20). In this pathway, the − 1 and − 2 z-score thresholds effectively separated pPD from oPD patients, with approximately three-quarters of subjects falling below the respective cutoffs appropriate for their disease stage. The LAH putamen/caudate ratio provided additional discriminative value in advanced stages, with three-quarters of oPD patients showing values below − 1.5, compared to less than one-quarter of prodromal subjects (Fig. 3, Table S8). This aligns with the expected floor effect in MAH putamen at advanced stages, where neurodegeneration in the initially affected hemisphere has already saturated this region, making the less-affected contralateral structures more informative about ongoing progression. A minority of oPD patients (7.4%) had a normal DaT-SPECT semiquantitative assessment but were all rated as abnormal at visual assessment, still considered the standard reference.

**Fig. 4:**
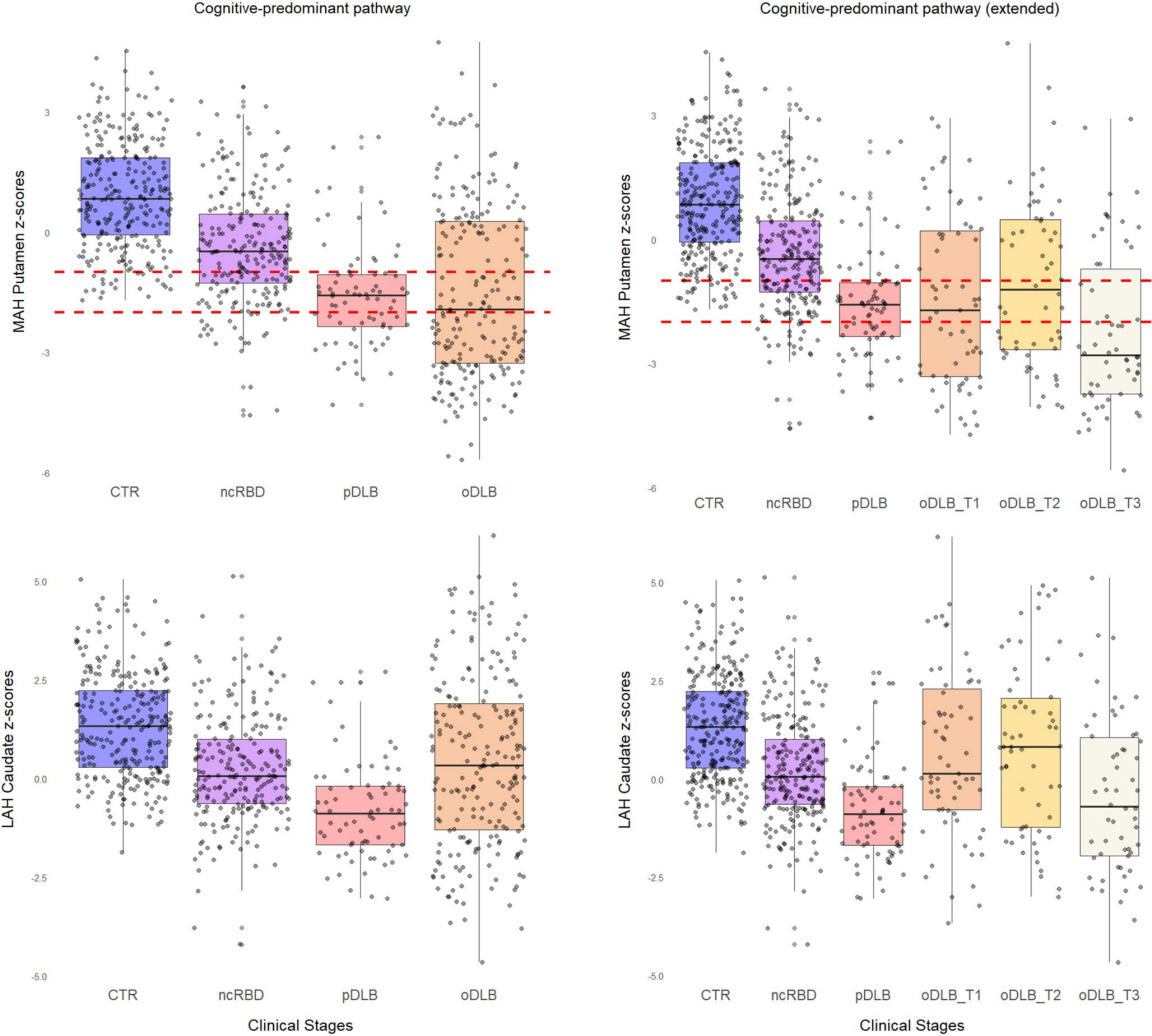
Presynaptic dopaminergic imaging features across the cognitive-predominant pathway. Box plot of the output of the two built models, representing the results for the cognitive-predominant pathway (left pane columns: controls [CTR], non-converters RBD [ncRBD], prodromal DLB [pDLB], and overt DLB [oDLB]) and cognitive-predominant extended pathway (right pane columns: controls, non-converters RBD [ncRBD], prodromal DLB [pDLB], and overt DLB, stratified in tertiles, in decreasing order of MMSE scores, oDLB-T3, oDLB-T2 and oDLB-T1, respectively). The top row shows the most affected putamen z-scores, and the dashed red lines represent the − 1 and − 2 z-scores cut-offs, respectively. The bottom row shows the least affected caudate z-scores for both models

The model assessing the association between DaT-SPECT features and the cognitive-predominant pathway (Fig. 4, Table S10) revealed that the best combination (*p* < 0.001) for predicting clinical stages includes the MAH putamen (OR 4.81, CI 3.82–6.19) and the LAH caudate (OR 0.26, CI 0.20–0.33). In this pathway, the MAH putamen z-score below − 1 captured most prodromal patients (77%), while the z-score below − 2 captured approximately half of overt DLB patients (49.3%), demonstrating substantially greater heterogeneity than observed in the motor-predominant pathway.

To investigate whether DaT-SPECT features could stage cognitive severity within oDLB, we performed a post-hoc analysis stratifying oDLB patients into tertiles according to MMSE scores. However, both the MAH putamen and LAH caudate showed floor effects across all oDLB tertiles, with no significant associations between DaT-SPECT features and cognitive severity within the overt DLB stage. This floor effect precludes using DaT-SPECT to stage cognitive severity within overt DLB. In contrast, in the motor-predominant pathway, LAH dopaminergic measures effectively track motor symptom progression, consistent with the motor manifestations of PD being primarily dopaminergic-mediated. The dissociation between dopaminergic pathology and cognitive severity in DLB aligns with evidence that cognitive decline in this disorder is primarily mediated by cholinergic system degeneration [26, 27].

To further explore the clinical significance of these cut-offs, we performed a post-hoc survival analysis in iRBD patients, using phenoconversion as the outcome. Survival time was defined as the time from DaT-SPECT to phenoconversion (for cRBD patients) or to last available follow-up (for ncRBD patients). Then, we performed a Cox regression analysis using the most affected putamen z-scores of −1 and − 2, and MDS-UPDRS-III, MMSE, and hyposmia as predictors. The hazard ratios (HRs) are summarized in Table 1.

**Table 1:** Results of the Cox regression analyses

	HR	95% CI
**Phenoconversion (whole sample)**		
Most affected Putamen z score − 1	3.10	2.10–4.57
Most affected Putamen z score − 2	3.74	2.68–5.23
MDS-UPDRS-III	2.60	1.87–3.62
MMSE	2.10	1.40–3.14
Hyposmia	2.72	1.67–4.44
**PD phenoconversion**		
Most affected Putamen z score − 1	3.49	2.09–5.80
Most affected Putamen z score − 2	5.03	3.18–7.96
MDS-UPDRS-III	3.09	1.98–4.83
MMSE	1.44	0.80–2.59
Hyposmia	2.58	1.37–4.85
**DLB phenoconversion**		
Most affected Putamen z score − 1	4.20	2.23–7.90
Most affected Putamen z score − 2	3.73	2.28–6.12
MDS-UPDRS-III	2.68	1.62–4.41
MMSE	4.40	2.46–7.89
Hyposmia	4.13	1.96–8.72

In brief, DaT-SPECT values showed higher HRs for predicting phenoconversion than clinical metrics in the whole sample and in the motor-predominant trajectory alone. In contrast, in the cognitive-predominant trajectory alone, MMSE showed the highest HR (4.40), exceeding both DaT-SPECT cutoffs.

Finally, post-hoc Spearman correlation revealed a strong, significant association between MAH putamen z-scores and MDS-UPDRS-III (rho = −0.38, *p* < 0.001).

## Discussion

This large, international, multicenter study aimed to define how DaT-SPECT should be used for staging neurodegeneration along the prodromal to overt neuronal alpha-synucleinopathy continuum at the individual level. Prior work has established DaT-SPECT as a phenoconversion risk marker in prodromal disease stages, but key questions remained about staging across the full disease spectrum and comparing DaT-SPECT with clinical metrics. DaT-SPECT is currently considered the most ready neurodegeneration biomarker for the nigro-striatal dopaminergic system [1, 2]. The role of DaT-SPECT as a phenoconversion risk factor in prodromal subjects was explored in a large, multicenter study conducted by the International RBD Study Group (IRBDSG), which demonstrated that visual assessment provides only moderate predictive ability [28]. A subsequent IRBDSG study showed that when scans are semi-quantified using a standardized approach, the DaT-SPECT prediction ability increases [25]. To deepen our understanding of the value of each basal ganglia region, the previous dataset was expanded and analyzed using advanced machine learning techniques, revealing that the most affected putamen best characterizes patients with RBD due to alpha-synucleinopathy [4]. However, several research questions remained open. First, it was unclear how DaT-SPECT could be used to stage neurodegeneration along the entire alpha-synucleinopathy continuum, including overt stages, at the individual level. Indeed, only a few single-center studies have assessed DaT-SPECT data across the whole prodromal-to-overt disease continuum [29–33]. Second, a head-to-head comparison between semi-quantified DaT-SPECT data and clinical risk factors for phenoconversion was missing. Finally, the current approach for assessing DaT-SPECT as abnormal or normal forces a progressive physiopathological process, namely neurodegeneration, into a strict binary categorization that likely does not reflect the complexity of the whole prodromal-to-overt disease continuum.

In the present IRBDSG study, we further expanded the previous dataset by including patients in overt stages, namely PD and DLB, as well as a large group of subjects without neurodegenerative disorders. We analyzed DaT-SPECT data, assuming progressive neurodegeneration levels from controls to non-converters RBD patients, converters RBD patients, and finally overt stage patients. We found that DaT-SPECT is significantly associated with all clinical stages along the clinical spectrum, with the most affected putamen showing the highest estimates in prodromal stages, and in the transition between prodromal and overt stages. Conversely, the least affected caudate showed an increased value in characterizing overt patients, especially those in the motor-predominant (parkinsonism-first) trajectory. This may seem in contradiction with the notion that nigro-caudate dopaminergic dysfunction is associated with cognitive impairment in PD [34–36]. However, it should be noted that here DaT-SPECT reflects the overall degree of the dopaminergic system neurodegeneration, which may not be strictly associated with all clinical signs. Indeed, in our study, the most affected DaT binding is significantly associated with MDS-UPDRS-III but not with MMSE scores.

In detail, our data suggest that the most affected putamen values can be used to define three levels of neurodegeneration at the individual level. That is, subjects with z-scores above − 1 are likely to be without evident neurodegeneration, subjects with values between − 1 and − 2 may be deemed to have moderate neurodegeneration, and subjects with z-scores below − 2 may be considered to have advanced neurodegeneration. This stratification is based on several lines of evidence. First, the most affected putamen z-score of −1 efficiently identifies iRBD patients at high risk of short-term phenoconversion. Notably, this cut-off reflects a high risk of overall phenoconversion (HR 3.10), of developing a parkinsonism-first phenotype (HR 3.49), as well as a dementia-first phenotype (HR 4.20). Interestingly, the semi-quantified DaT-SPECT features outperform the clinical risk factors for overall and motor-predominant phenoconversion. However, in the cognitive-predominant trajectory, MMSE showed similar or slightly higher predictive values than DaT-SPECT cutoffs. The most affected putamen z-score of −2 further increases the risk of phenoconversion, especially in the motor-predominant trajectory. It is worth highlighting that this cut-off characterizes most overt PD patients, while it identifies about half of overt DLB patients. This is consistent with the notion that the dopaminergic system does not solely mediate the decline in cognition in neuronal alpha-synucleinopathies, which is primarily mediated by the cholinergic system [26, 27]. Thus, DLB patients showing a wide heterogeneity at both putamen and caudate levels is not unexpected. Notably, we enrolled DLB patients without further stratification. That is, we may have included patients with and without RBD, as well as patients with and without parkinsonism. Unfortunately, we don’t have more detailed information to perform a deeper phenotypization, which is a limitation of the present study. Thus, our data suggest that DaT-SPECT can assess nigro-striatal neurodegeneration in the prodromal to overt alpha-synucleinopathy continuum, including iRBD patients eventually phenoconverting to both PD and DLB. Conversely, additional metrics are needed to stage neurodegeneration in overt DLB patients.

**Fig. 5:**
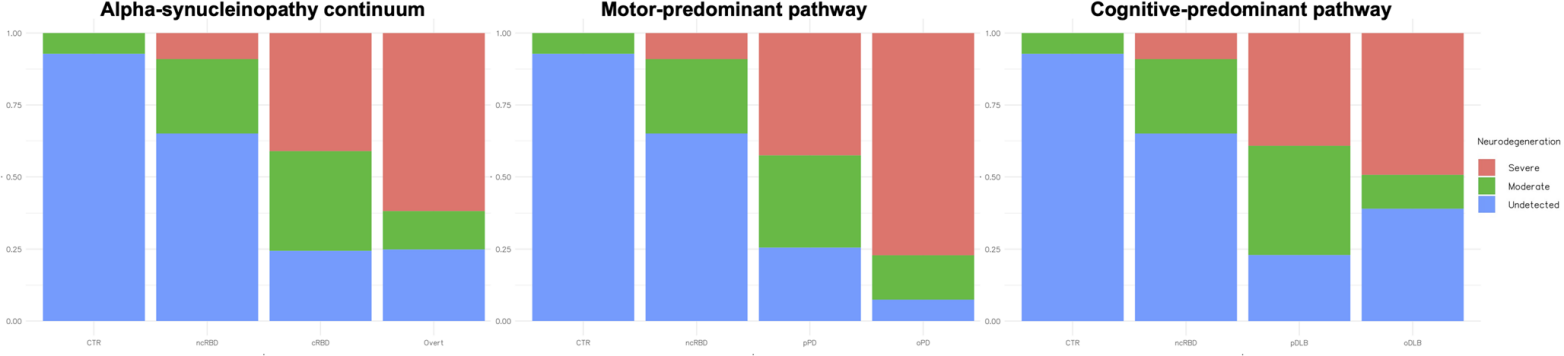
Dopaminergic neurodegeneration levels across the prodromal to overt alpha-synucleinopathy continuum. Bar charts showing the three-tier neurodegeneration levels (undetected, moderate, and severe) across the whole alpha-synucleinopathy continuum, the motor-predominant (parkinsonism-first) pathway, and the cognitive predominant (dementia-first) pathway. The proposed three-tier neurodegeneration staging system, based on the most affected putamen DaT-SPECT z-scores, characterizes the progressive neurodegeneration levels of the study cohorts, including subjects in the prodromal alpha-synucleinopathy stages

Current research frameworks for defining PD and related disorders only envision a simplistic binary model of positive and negative neurodegeneration biomarkers [1, 2]. This is a limitation of the current proposal, mainly because the clinical milestones identify at least two stages, namely prodromal and overt. Thus, neurodegeneration biomarkers should be able to provide at least three levels of neurodegeneration, such as undetected, moderate, and severe, to parallel clinical stages. Notably, it is not expected that the neurodegeneration levels identified by using functional neuroimaging techniques, such as DaT-SPECT, perfectly align with the clinical stages. Indeed, several compensatory mechanisms, including physical reserve and genetically determined resilience, modulate the clinical impact of neurodegeneration [37, 38]. Nevertheless, the proposed neurodegeneration levels, with the respective cut-offs, have evident clinical correlates, in terms of outcome prediction, but also by characterizing patients in prodromal (moderate neurodegeneration) versus overt (severe neurodegeneration) stages, especially those in the motor-predominant trajectory. Thus, from a practical perspective, we envision a staging system that categorizes patients as having undetected, moderate, or severe neurodegeneration, regardless of the clinical stage. This is a new and modern approach that allows describing patients’ neurodegenerative levels with more detailed, clinically relevant stages, fulfilling the need to adopt personalized precision medicine (Fig. 5).

Focusing on our overt stage patients, we found that the least affected caudate best tracked the clinical disease progression, especially in the motor-predominant pathway. This result aligns well with the hypothesis that nigro-striatal neurodegeneration typically begins in one putamen (i.e., in the most affected hemisphere) and subsequently spreads to the other regions, with the contralateral caudate (i.e., in the least affected hemisphere) being the last nucleus to become involved, at least in PD [39]. Thus, while the most affected putamen is expected to be the most relevant feature in early stages, its progressive neurodegeneration causes a floor effect in advanced stages, where the least affected caudate likely retains most of explained variance. This result is in line with a recent study on a large dataset of overt PD patients, showing that the best progression DaT-SPECT feature is in the least affected hemisphere. [9]. Overall, these findings suggest that DaT-SPECT may be an efficient objective biomarker for tracking disease progression along the prodromal to overt alpha-synucleinopathy continuum. However, a large longitudinal study, possibly including all clinical stages and trajectories is needed to definitely answer this question.

This study has strengths and limitations. The main strength is the unprecedentedly large sample size, including patients at every clinical stage along the alpha-synucleinopathy continuum. Moreover, DaT-SPECT data were semi-quantified using a standardized, easily reproducible approach, making the results applicable in clinical practice at the individual level. The main limitation is the lack of longitudinal imaging data needed to assess DaT-SPECT as a disease progression monitoring biomarker properly. Moreover, our prodromal cohort includes only iRBD patients, whereas other subjects, such as those with hyposmia or asymptomatic PD-related genetic mutations, were not included in this study. Furthermore, the duration of the symptoms before diagnosis was missing. Finally, the clinical markers included in the present study assessed only motor and cognitive symptoms; other non-motor markers may have been of interest. However, a more detailed clinical assessment was beyond the aim of the present study. Unfortunately, Alzheimer’s disease-related co-pathology biomarkers were not available in this study as well.

In conclusion, our study supports the use of DaT-SPECT as a staging neurodegeneration biomarker at the individual level along the prodromal to overt alpha-synucleinopathy continuum. We propose an operational approach using three z-score thresholds for staging neurodegeneration based on DaT-SPECT (>−1: undetected; −1 to −2: moderate; <−2: severe), providing clear, quantitative thresholds for individual patient staging. If implemented in future revisions of alpha-synucleinopathy staging systems, these cutoffs would enable clinicians to objectively stage neurodegeneration severity, stratify phenoconversion risk in prodromal patients, and monitor disease trajectory in established disease. This approach should be tested in a longitudinal DaT-SPECT study to determine whether changes in clinical stages consistently align with changes in neurodegenerative levels over time.

## Supplementary Information

Below is the link to the electronic supplementary material.

Supplementary Material 1 (DOCX 34.9 KB)

## Data Availability

The datasets generated during and/or analysed during the current study are available from the corresponding author on reasonable request.
