# Supplementary material for: Presynaptic dopaminergic imaging as a neurodegeneration staging biomarker in the alpha-synucleinopathy continuum
Source: Eur J Nucl Med Mol Imaging. 2026 Apr 1;53(7):4677–88. doi: 10.1007/s00259-026-07834-1 (PMC13197272; doi:10.1007/s00259-026-07834-1)
Supplement: Supplementary file 1 — Supplementary Material 1 (DOCX 34.9 KB) [file 259_2026_7834_MOESM1_ESM.docx]

**Supplementary material**

Supplementary Table 1. Main clinical and demographic characteristic of the sample, according to the center belonging

|  | **Barcelona (N=157)** | **Berlin (N=12)** | **Bologna (N=15)** | **Cagliari (N=44)** | **Chieti (N=61)** | **Dokkyo (N=28)** | **Geneve (N=100)** | **Genoa (N=338)** | **Kosice (N=8)** | **MCR (N=37)** | **Montpellier (N=22)** | **Oxford (N=8)** | **Pavia (N=20)** | **Prague (N=173)** | **Tor Vergata (N=44)** | **Total (N=1067)** |
| --- | --- | --- | --- | --- | --- | --- | --- | --- | --- | --- | --- | --- | --- | --- | --- | --- |
| AGE |  |  |  |  |  |  |  |  |  |  |  |  |  |  |  |  |
| - Mean (SD) | 68.27 (7.15) | 63.58 (8.61) | 65.93 (7.62) | 70.82 (7.08) | 65.57 (12.40) | 69.57 (4.12) | 77.70 (6.76) | 71.78 (8.01) | 69.38 (7.91) | 65.86 (8.79) | 68.59 (7.42) | 72.25 (5.15) | 66.60 (7.88) | 66.54 (8.53) | 67.43 (7.99) | 69.78 (8.73) |
| - Median (Q1, Q3) | 68.00 (64.00, 73.00) | 61.50 (57.25, 72.25) | 67.00 (62.00, 70.00) | 70.00 (66.00, 76.25) | 68.00 (60.00, 74.00) | 70.00 (66.00, 73.00) | 78.50 (73.75, 83.00) | 73.00 (67.00, 78.00) | 71.00 (69.00, 73.00) | 65.00 (60.00, 72.00) | 67.50 (63.50, 73.75) | 73.50 (70.00, 75.25) | 66.50 (61.25, 71.00) | 66.71 (60.76, 73.44) | 68.00 (62.00, 71.25) | 70.34 (64.00, 76.00) |
| SEX |  |  |  |  |  |  |  |  |  |  |  |  |  |  |  |  |
| - Female | 45 (28.7%) | 1 (8.3%) | 2 (13.3%) | 13 (29.5%) | 26 (42.6%) | 6 (21.4%) | 41 (41.0%) | 154 (45.6%) | 3 (37.5%) | 10 (27.0%) | 5 (22.7%) | 1 (12.5%) | 5 (25.0%) | 59 (34.1%) | 22 (50.0%) | 393 (36.8%) |
| - Male | 112 (71.3%) | 11 (91.7%) | 13 (86.7%) | 31 (70.5%) | 35 (57.4%) | 22 (78.6%) | 59 (59.0%) | 184 (54.4%) | 5 (62.5%) | 27 (73.0%) | 17 (77.3%) | 7 (87.5%) | 15 (75.0%) | 114 (65.9%) | 22 (50.0%) | 674 (63.2%) |
| DIAGNOSIS |  |  |  |  |  |  |  |  |  |  |  |  |  |  |  |  |
| - CTR | 12 (7.6%) | 6 (50.0%) | 5 (33.3%) | 10 (22.7%) | 0 (0.0%) | 7 (25.0%) | 0 (0.0%) | 163 (48.2%) | 2 (25.0%) | 6 (16.2%) | 4 (18.2%) | 6 (75.0%) | 5 (25.0%) | 36 (20.8%) | 15 (34.1%) | 277 (26.0%) |
| - DLB | 0 (0.0%) | 0 (0.0%) | 0 (0.0%) | 0 (0.0%) | 61 (100.0%) | 0 (0.0%) | 100 (100.0%) | 54 (16.0%) | 0 (0.0%) | 0 (0.0%) | 0 (0.0%) | 0 (0.0%) | 0 (0.0%) | 0 (0.0%) | 0 (0.0%) | 215 (20.1%) |
| - PD | 0 (0.0%) | 0 (0.0%) | 0 (0.0%) | 0 (0.0%) | 0 (0.0%) | 0 (0.0%) | 0 (0.0%) | 75 (22.2%) | 0 (0.0%) | 0 (0.0%) | 0 (0.0%) | 0 (0.0%) | 0 (0.0%) | 100 (57.8%) | 0 (0.0%) | 175 (16.4%) |
| - iRBD | 145 (92.4%) | 6 (50.0%) | 10 (66.7%) | 34 (77.3%) | 0 (0.0%) | 21 (75.0%) | 0 (0.0%) | 46 (13.6%) | 6 (75.0%) | 31 (83.8%) | 18 (81.8%) | 2 (25.0%) | 15 (75.0%) | 37 (21.4%) | 29 (65.9%) | 400 (37.5%) |
| MDS=UPDRS=III |  |  |  |  |  |  |  |  |  |  |  |  |  |  |  |  |
| - Mean (SD) | 2.42 (2.93) | 2.33 (3.21) | 2.30 (1.89) | 0.94 (1.23) | 20.58 (9.86) | 2.90 (2.17) | 26.83 (15.51) | 14.57 (12.38) | 6.50 (4.37) | 1.71 (2.42) | 3.61 (3.93) | 6.50 (2.12) | 2.27 (2.15) | 23.66 (15.28) | 2.93 (2.34) | 12.78 (14.27) |
| - Median (Q1, Q3) | 1.00 (0.00, 4.00) | 1.00 (0.50, 3.50) | 2.00 (0.50, 4.00) | 0.00 (0.00, 1.75) | 17.00 (13.00, 26.00) | 2.00 (2.00, 4.00) | 26.00 (14.50, 35.00) | 13.00 (3.00, 23.00) | 6.50 (3.50, 8.75) | 1.00 (0.00, 3.00) | 2.50 (1.00, 3.75) | 6.50 (5.75, 7.25) | 3.00 (0.00, 3.50) | 24.00 (10.00, 35.00) | 3.00 (0.00, 5.00) | 6.00 (1.75, 22.00) |
| MMSE |  |  |  |  |  |  |  |  |  |  |  |  |  |  |  |  |
| - Mean (SD) | 27.72 (2.34) | 29.00 (0.00) | 27.55 (1.06) | 25.88 (4.36) | 20.64 (2.75) | 27.76 (2.26) | 20.07 (4.86) | 26.60 (3.42) | 29.00 (0.89) | 28.23 (1.78) | 29.00 (1.64) | 27.00 (1.41) | 27.53 (2.77) | 27.05 (1.99) | 26.60 (1.84) | 25.84 (4.22) |
| - Median (Q1, Q3) | 28.00 (27.00, 29.00) | 29.00 (29.00, 29.00) | 28.00 (27.25, 28.23) | 28.00 (26.00, 28.00) | 21.00 (19.00, 23.00) | 29.00 (26.00, 30.00) | 21.00 (17.00, 23.25) | 28.00 (25.00, 29.00) | 29.00 (28.25, 29.75) | 29.00 (27.00, 30.00) | 30.00 (28.25, 30.00) | 27.00 (26.50, 27.50) | 28.00 (26.50, 30.00) | 28.00 (26.00, 28.00) | 26.30 (25.40, 28.00) | 27.00 (24.00, 29.00) |

Table S2. Main clinical and demographic data of the whole cohort, stratified according to 3-staging levels (controls, prodromal and overt stage). Continuous data are shown as mean ± standard deviation (median), while categorical variables are shown as percentage.

|  | **3-staging levels** | | | |
| --- | --- | --- | --- | --- |
|  | Controls  (277) | Prodromal stage  (400) | Overt stage  (390) | p value |
| Age, years | 68.3±9.0 (69) | 68.9±6.9 (69) | 71.7±9.9 (73) | <0.001 |
| Sex, males | 50.2% | 75.2% | 60% | <0.001 |
| MDS-UPDRS-III | N/A | 2.6±3.2 (2) | 24.3±13.2 (23) | <0.001 |
| MMSE | N/A | 27.6±2.5 (28) | 23.2±4.9 (24) | <0.001 |

Legend: MDS-UPDRS-III, movement disorder society unified Parkinson disease rating scale, motor section; MMSE, mini-mental state examination test. P-values from ANOVA/Kruskal-Wallis or chi-square tests as appropriate (cf. manuscript).

Table S3. Main clinical and demographic data of the whole cohort, stratified according to 4-staging levels (controls, non-converters RBD, [ncRBD], converters RBD, [cRBD], and overt stage). Continuous data are shown as mean ± standard deviation (median), while categorical variables are shown as percentage.

|  | **4-staging levels** | | | | |
| --- | --- | --- | --- | --- | --- |
|  | Controls  (277) | ncRBD  (232) | cRBD  (168) | Overt stage  (390) | p value |
| Age, years | 68.3±9.0 (69) | 67.6±7.1 (68) | 70.8±6.1 (71) | 71.7±9.9 (73) | <0.001 |
| Sex, males | 50.2% | 78.4% | 70.8% | 60% | <0.001 |
| MDS-UPDRS-III | N/A | 1.8±2.5 (1) | 3.8±3.8 (3) | 24.3±13.2 (23) | <0.001 |
| MMSE | N/A | 28.0±2.4 (29) | 27.1±2.5 (28) | 23.2±4.9 (24) | <0.001 |

Legend: MDS-UPDRS-III, movement disorder society unified Parkinson disease rating scale, motor section; MMSE, mini-mental state examination test.

Table S4. Main clinical and demographic data of the whole cohort, stratified according to the motor-predominant pathway (controls, non-converters RBD, [ncRBD], prodromal PD, [pPD], and overt PD, [oPD). Continuous data are shown as mean ± standard deviation (median), while categorical variables are shown as percentage.

|  | **Motor-predominant pathway** | | | | |
| --- | --- | --- | --- | --- | --- |
|  | Controls  (277) | ncRBD  (232) | pPD  (94) | oPD  (175) | p value |
| Age, years | 68.3±9.0 (69) | 67.6±7.1 (68) | 69.7±5.8 (69) | 68.7±8.6 (70) | <0.001 |
| Sex, males | 50.2% | 78.4% | 61.7% | 60% | <0.001 |
| MDS-UPDRS-III | N/A | 1.8±2.5 (1) | 4.5±4.4 (4) | 25.9±11.9 (25) | <0.001 |
| MMSE | N/A | 28.0±2.4 (29) | 27.4±2.5 (28) | 27.7±2.5 (28) | <0.001 |

Legend: MDS-UPDRS-III, movement disorder society unified Parkinson disease rating scale, motor section; MMSE, mini-mental state examination test.

Table S5. Main clinical and demographic data of the whole cohort, stratified according to the cognitive-predominant pathway (controls, non-converters RBD, [ncRBD], prodromal DLB, [pDLB], and overt DLB, [oDLB]). Continuous data are shown as mean ± standard deviation (median), while categorical variables are shown as percentage.

|  | **Cognitive-predominant pathway** | | | | |
| --- | --- | --- | --- | --- | --- |
|  | Controls  (277) | ncRBD  (232) | pDLB  (74) | oDLB  (215) | p value |
| Age, years | 68.3±9.0 (69) | 67.6±7.1 (68) | 72.1±6.1 (72) | 74.2±10.2 (76) | <0.001 |
| Sex, males | 50.2% | 78.4% | 82.4% | 60% | <0.001 |
| MDS-UPDRS-III | N/A | 1.8±2.5 (1) | 2.9±2.9 (2) | 22.6±14.2 (20) | <0.001 |
| MMSE | N/A | 28.0±2.4 (29) | 26.7±2.4 (27) | 21.3±4.4 (22) | <0.001 |

Legend: MDS-UPDRS-III, movement disorder society unified Parkinson disease rating scale, motor section; MMSE, mini-mental state examination test.

Table S6. Most affected hemisphere (MAH) putamen z-scores and least affected hemisphere (LAH) caudate z-scores across the 3-staging levels (Controls, prodromal and overt stages).

|  | **3-staging levels** | | |
| --- | --- | --- | --- |
|  | Controls  (277) | Prodromal stage  (400) | Overt stage  (390) |
| MAH putamen, z-scores  -Mean ± standard deviation  -Median (Q1, Q3) | 0.9 ± 1.3  0.8 (-0.1, 1.9) | -0.9 ± 1.5  -1.1 (-1.9, -0.1) | -2.0 ± 1.8  -2.5 (-3.2, -1.1) |
| LAH caudate, z-scores  -Mean ± standard deviation  -Median (Q1, Q3) | 1.3 ± 1.3  1.3 (0.3, 2.2) | -0.2 ± 1.4  -0.3 (-1.1, 0.6) | -0.1 ± 1.9  -0.3 (-1.4, 1.1) |

Table S7. Most affected hemisphere (MAH) putamen z-scores and least affected hemisphere (LAH) caudate z-scores across the 4-staging levels (Controls, non-converters RBD, [ncRBD], converters RBD, [cRBD], and overt stage).

|  | **4-staging levels** | | | |
| --- | --- | --- | --- | --- |
|  | Controls  (277) | ncRBD  (232) | cRBD  (168) | Overt  (390) |
| MAH putamen, z-scores  -Mean ± standard deviation  -Median (Q1, Q3) | 0.9 ± 1.3  0.8 (-0.1, 1.9) | -0.4 ± 1.4  -0.5 (-1.3, 0.5) | -1.7 ± 1.3  -1.7 (-2.6, -1.0) | -2.0 ± 1.8  -2.5 (-3.2, -1.1) |
| LAH caudate, z-scores  -Mean ± standard deviation  -Median (Q1, Q3) | 1.3 ± 1.3  1.3 (0.3, 2.2) | 0.2 ± 1.4  0.1 (-0.6, 1.0) | -0.8 ± 1.2  -0.8 (-1.6, -0.1) | -0.1 ± 1.9  -0.3 (-1.4, 1.1) |

Table S8. Most affected hemisphere (MAH) putamen z-scores and least affected hemisphere (LAH) putamen/caudate (P/C) ratio z-scores across the motor-predominant pathway (Controls, non-converters RBD [ncRBD], prodromal P, [pPD], and overt PD [oPD]).

|  | **Motor predominant pathway** | | | |
| --- | --- | --- | --- | --- |
|  | Controls  (277) | ncRBD  (232) | pPD  (94) | oPD  (175) |
| MAH putamen, z-scores  -Mean ± standard deviation  -Median (Q1, Q3) | 0.9 ± 1.3  0.8 (-0.1, 1.9) | -0.4 ± 1.4  -0.5 (-1.3, 0.5) | -1.8 ± 1.3  -1.8 (-2.6, -1.0) | -2.6 ± 0.9  -2.7 (-3.2, -2.1) |
| LAH P/C ratio, z-scores  -Mean ± standard deviation  -Median (Q1, Q3) | -0.1 ± 1.0  -0.1 (-0.7, 0.6) | -0.3 ± 1.2  -0.3 (-1.2, 0.5) | -0.6 ± 1.5  -0.7 (-1.4, 0.3) | -2.3 ± 1.8  -2.4 (-3.4, -1.5) |

Table S9. Most affected hemisphere (MAH) putamen z-scores and least affected hemisphere (LAH) putamen/caudate (P/C) ratio z-scores across the motor-predominant extended pathway (controls, non-converters RBD [ncRBD], prodromal PD [pPD], and overt PD with Hoehn and Yahr scale score of 1, 2 and 3, respectively).

|  | **Motor predominant pathway extended** | | | | | |
| --- | --- | --- | --- | --- | --- | --- |
|  | Controls  (277) | ncRBD  (232) | pPD  (94) | H&Y 1  (28) | H&Y 2  (131) | H&Y 3  (16) |
| MAH putamen, z-scores  -Mean ± SD  -Median (Q1, Q3) | 0.9±1.3  0.8 (-0.1, 1.9) | -0.4 ± 1.4  -0.5 (-1.3, 0.5) | -1.8 ± 1.3  -1.8 (-2.6, -1.0) | -2.5 ± 0.8  -2.6 (-3.1, -2.0) | -2.6 ± 0.9  -2.6 (-3.3, -2.1) | -2.5 ± 1.1  -2.8 (-3.0, -2.0) |
| LAH P/C ratio, z-scores  -Mean ± SD  -Median (Q1, Q3) | 1.3 ± 1.3  1.3 (0.3, 2.2) | 0.2 ± 1.4  0.1 (-0.6, 1.0) | -0.7 ± 1.2  -0.6 (-1.5, 0.1) | -0.3 ± 1.5  -0.4 (-1.4, 0.6) | -0.6 ± 1.3  -0.8 (-1.5, 0.1) | -1.1 ± 1.4  -1.0 (-2.1, -0.6) |

Table S10. Most affected hemisphere (MAH) putamen z-scores and least affected hemisphere (LAH) caudate z-scores across the cognitive-predominant pathway (Controls, non-converters RBD, [ncRBD], prodromal DLB, [pDLB], and overt DLB, [oDLB]).

|  | **Cognitive predominant pathway** | | | |
| --- | --- | --- | --- | --- |
|  | Controls  (277) | ncRBD  (232) | pDLB  (74) | oDLB  (215) |
| MAH putamen, z-scores  -Mean ± standard deviation  -Median (Q1, Q3) | 0.9 ± 1.3  0.8 (-0.1, 1.9) | -0.4 ± 1.4  -0.5 (-1.3, 0.5) | -1.6 ± 1.3  -1.6 (-2.3, -1.0) | -1.5 ± 2.2  -1.9 (-3.3, -0.3) |
| LAH caudate, z-scores  -Mean ± standard deviation  -Median (Q1, Q3) | 1.3 ± 1.3  1.3 (0.3, 2.2) | 0.2 ± 1.4  0.1 (-0.6, 1.0) | -0.8 ± 1.2  -0.9 (-1.7, -0.2) | 0.4 ± 2.2  0.3 (-1.3, 1.9) |

Table S11. Most affected hemisphere (MAH) putamen z-scores and least affected hemisphere (LAH) caudate z-scores across the cognitive-predominant extended pathway (Controls, non-converters RBD, [ncRBD], prodromal DLB, [pDLB], and overt DLB, stratified in tertiles, in decreasing order of MMSE scores, [oDLB-T3], [oDLB-T2] and [oDLB-T1], respectively).

|  | **Cognitive predominant pathway extended** | | | | | |
| --- | --- | --- | --- | --- | --- | --- |
|  | Controls  (277) | ncRBD  (232) | pDLB  (74) | oDLB-T3  (58) | oDLB-T2  (58) | oDLB-T1  (63) |
| MAH putamen, z-scores  -Mean ± SD  -Median (Q1, Q3) | 0.9±1.3  0.8 (-0.1, 1.9) | -0.4 ± 1.4  -0.5 (-1.3, 0.5) | -1.6 ± 1.3  -1.6 (-2.3, -1.0) | -2.2 ± 1.9  -2.8 (-3.7, -0.7) | -1.0 ± 2.0  -1.2 (-2.7, 0.5) | -1.5 ± 2.1  -1.7 (-3.3, 0.2) |
| LAH caudate, z-scores  -Mean ± SD  -Median (Q1, Q3) | 1.3 ± 1.3  1.3 (0.3, 2.2) | 0.2 ± 1.4  0.1 (-0.6, 1.0) | -0.8 ± 1.2  -0.9 (-1.7, -0.2) | -0.4 ± 2.0  -0.7 (-1.9, 1.1) | 0.8 ± 2.2  0.8 (-1.2, 2.1) | 0.6 ± 2.1  0.1 (-0.8, 2.3) |
